# Monitoring Antiepileptic Drugs: A Level-Headed Approach

**DOI:** 10.2174/157015909788848938

**Published:** 2009-06

**Authors:** Erik K St. Louis

**Affiliations:** Mayo Clinic, Rochester, Minnesota, USA

**Keywords:** Epilepsy, antiepileptic drugs, blood levels, monitoring.

## Abstract

Despite advances in epilepsy therapeutics, some physicians feel uncomfortable with newer antiepileptic drugs (AEDs) due to difficulty in promptly obtaining blood levels to guide medication adjustment, and even when levels for newer AEDs are obtained, many practitioners feel they are not very useful. Lacking confidence in AEDs whose levels that cannot readily or expeditiously be measured, many clinicians share uncertainty about proper use of the newer AEDs and monitoring AED administration. Similarly, some epilepsy patients inflate the importance of AED blood level monitoring, feeling that blood levels falling within traditionally therapeutic ranges are a fail-safe for seizure control, regardless of their compliance or personal behavior aggravating seizure burden, such as poor sleep or use of illicit substances. This review examines the elusive concept of therapeutic AED blood levels and potential uses and abuses of blood level monitoring, reinforcing appropriate uses for blood levels to ensure compliance and adjust for altered AED pharmacokinetics in the context of aging and disease states, pregnancy, or drug interactions.

## NO HOME ON THE THERAPEUTIC RANGE: THE MYTHOS OF AED BLOOD LEVEL MONITORING

The imperative of maintaining AEDs within usual laboratory defined therapeutic ranges is a myth subject to longstanding criticism [1,2,8]. While there is evidence for older AEDs blood level ranges producing therapeutic effects for most patients and toxic threshold levels for many, [9,11] laboratory therapeutic ranges word defined in populations. When considering the blood level obtained in an individual patient there may be considerable variability in response. No definite correlation between a patient’s specific blood level and clinical efficacy and toxicity may exist, even in the use of the older AEDs, but especially with the newer AEDs where blood level ranges of efficacy and tolerability have often shown wide variability and inconsistent results.

## PROBLEMS OF MONITORING EFFICACY WITH LEVELS

AED blood levels in a seizure-free patient may be well above or below the standard laboratory therapeutic range, especially when high dose monotherapy is necessary for seizure control where levels may rise well into the “toxic” range to control seizures in intractable patients [7]. Likewise, lower or “subtherapeutic” blood levels may control seizures in many patients, and raising the dose to “treat the level” and bring it into the therapeutic range does not necessarily improve seizure control [13]. Whether AED blood level monitoring enhances the clinical wisdom of maximally tolerable dosing toward seizure freedom remains unclear [6]. A prospective study of AED blood level monitoring with older AEDs (for which there is most agreement of the validity of established therapeutic ranges) found no difference in outcomes of reported seizure control or adverse effects between patients randomized to AED adjustment by clinical practice, or those who received AED therapy directed to achieve target blood levels [4].

The historical dictum for achieving optimally efficacious AED therapy in refractory epilepsy has suggested titration to a maximally tolerable dosage, usually resulting in robust blood levels. It has been generally believed that to ensure an adequate therapeutic trial, AED doses should be pushed to a toxic threshold prior to abandoning an AED and considering future adjunctive therapy [14]. While blood levels may be desirable for documenting an adequate therapeutic trial and ensuring compliance, particularly for intractable patients pursuing presurgical evaluation, recent prospective observational research has called the practice of maximal tolerated dosing into question. An evolving perspective toward the use of moderate AEDs dosages may be reasonable since moderate dose ranges appear effective and tolerable for the vast majority of medically responsive patients [5]. Further research is needed to confirm the “moderate dose approach” to AED therapy and to define what, if any, additional role exists for AED blood level determination.

## PROBLEMS OF MONITORING TOXICITY WITH BLOOD LEVELS

Conversely, clinically significant toxic adverse effects may develop even at low doses and levels in many patients, so that there is no “safety net” at the bottom of AED therapeutic ranges for ensuring patient tolerability. Given insensitivity of AED blood level monitoring to subjective adverse effects, an expeditious bedside tool could heighten clinician vigilance for the identification of patients experiencing adverse effects and lead to more proactive AED adjustment. A recent study systematically screened epilepsy outpatients at risk for AED adverse effects and found that the Adverse Events Profile, a patient checklist for subjective perceived AED adverse effects, increased identification of AED toxicity and guided appropriate adjustment of therapy without loss of seizure control [3]. Future studies correlating the AEP and blood levels are necessary to determine which is better for identifying AED adverse effects; the AEP is certainly a cheaper and more expeditious method.

A practical issue in obtaining levels in the “therapeutic” window is that variability in the time of measurement and AED co-therapy influences AED blood levels. Morning trough levels are felt to most accurately represent whether the patient is protected against seizure occurrence, but many times morning trough levels are logistically difficult to obtain without planning and advance discussion with the patient. Measuring levels during the window of the patient’s perceived adverse effects may be more desirable when determining toxicity threshold for an AED. When measuring levels of AEDs with very short half-lives, such as carbamazepine with an enzyme-inducing AED as co-therapy, multiple level measurements over the course of several hours may help establish the curve of peak-trough variation and subsequently guide dosing interval adjustments.

## ABUSES OF AED BLOOD LEVEL MONITORING

Both physicians and patients alike sometimes ignore the longstanding maxim of AED therapy to “treat the patient, not the level” [1,14]. AED blood levels should always be considered as complimentary and subservient to the paramount treatment goals of achieving seizure-freedom and maximizing quality of life. When AED therapy is predicated solely on preserving “therapeutic” blood levels, two inappropriate courses of action can result; complacency in the face of ongoing intractable seizures, or perhaps even worse, injudicious adjustment of AED therapy when levels are perceived as “subtherapeutic” or “toxic” in a seizure-free patient. Such approaches may inappropriately shift the focus of treatment from the patient to the laboratory, from attaining seizure freedom and maximal quality of life to a preoccupation with attaining “therapeutic” levels. Table **1** reviews these potential pitfalls in clinical application of AED blood levels.

## USES OF AED BLOOD LEVEL MONITORING

There are many perfectly appropriate uses of AED blood level monitoring that should be capitalized upon. Appropriate uses of AED blood levels include: assisting with AED conversions; ensuring adherence or compliance; elucidating pharmacokinetics of an AED with a complex pharmacology; and adjusting for altered AED pharmacokinetics consequent to aging, disease states, drug interactions, and pregnancy. These typical uses are now considered in further detail below.

## ASSISTING WITH AED CONVERSIONS

AED blood level monitoring can be especially helpful following initiation of a new AED therapy, either in denovo monotherapy, or in guiding conversions between monotherapies or when a new AED is added into a complex polytherapy regimen. AED blood levels can help guide titration of the AED toward a target dose-providing efficacy for most patients, helping to ensure that the patient has reached a sufficiently protective dosage of a newly administered AED. Blood level monitoring can assist titration of new adjunctive AEDs in complex polypharmacy regimens, when drug interactions may influence either the new adjunctive drug, or the baseline AED regimen and other concurrent medications. Blood levels may also help to determine which AED is most responsible for adverse effects in a patient receiving polytherapy.

## ENSURING ADHERENCE OR COMPLIANCE

Some clinicians find value in serially measuring blood levels to ensure optimal adherence or compliance. The value of blood level determination in enhancing compliance has been demonstrated [10]. However, the cost of regular serial blood level monitoring in all epilepsy patients would become prohibitive to the health system if regularly applied in all epilepsy patients. For most patients, single blood level determination during successful maintenance AED therapy may be beneficial to serves as an “individual benchmark” for future comparison if seizure control deteriorates, and the clinician is questioning whether therapeutic failure is due to non-compliance, changes in AED pharmacokinetics, or evolution of medical intractability. Blood level measurement when patients become medically intractable also has utility given inter-individual variability in AED responsiveness.

## ELUCIDATING PHARMACOKINETICS OF AN AED WITH A COMPLEX PHARMACOLOGY

Phenytoin is an example of the inherent value of blood level monitoring when AEDs have complex pharmacokinetics. Phenytoin blood levels may assist in avoiding therapeutic overshoot of dosing and development of overt clinical toxicity, given saturable metabolism seen in higher phenytoin dose ranges. Carbamazepine-10,11-epoxide levels may be helpful in selected patients to confirm vague clinically toxic side effects occurring at low to moderate total serum levels, especially in polytherapy regimens.

## ADJUSTING FOR ALTERED AED PHARMACOKINETICS

AED levels should be measured during physiologic alterations due to aging, diseases, drug interactions, and pregnancy. As a child ages through the first decade of life and especially nearing puberty, drug metabolism and clearance may increase dramatically, requiring increased AED doses to maintain a weight-based steady state. At the other extreme, elderly may have decreases in drug metabolism and clearance necessitating lower AED dosages. Pregnancy causes dramatic changes in drug pharmacokinetics and leads to altered drug absorption, metabolism, protein binding and clearance, so that serial levels before pregnancy, during each trimester, and during the postpartum state may help guide appropriate dose augmentation or reduction. AED blood levels are especially helpful in disease states that alter AED pharmacokinetics, such as renal disease. With some heavily protein-bound drugs, especially phenytoin, obtaining free drug levels is necessary to discern the biologically active fraction of the drug, especially in conditions where drugs are competitively displaced from binding to plasma protein, such as in chronically or critically ill patients with uremia or hypoalbuminemia. Polypharmacy is another indication for blood level determination, especially with complex AED interactions involving hepatic enzyme induction or inhibition. Table **2** depicts clinical scenarios where AED blood level monitoring may be particularly helpful, and lists common usual courses of action.

## NEWER AED BLOOD LEVELS AND SAFETY MONITORING

Newer AED blood levels are not widely or immediately available, and at many centers are “send-out” tests that may not return for review and correlation with the clinical context for weeks. There is considerably less data regarding usually effective and toxic blood level ranges with newer AEDs, but practical experience and expert opinion have suggested useful ranges. Table **3** provides a practical guide to usual effective doses and blood levels for the older and newer AEDs.

## APPROPRIATE BLOOD LEVEL MONITORING PRACTICES AND AED ADJUSTMENTS

Table **4** provides usual strategies for AED dosing adjustments in the context of a patient’s clinical seizure control and adverse effect symptoms. While some patients develop breakthrough seizures even at supratherapeutic or “toxic” levels, others may experience adverse effects within the usual “therapeutic” range, while some patients will become seizure-free on levels in a “subtherapeutic” range.

A potential mismatch between a patient’s clinical status and blood levels may create potential problems if the clinician over relies on blood level monitoring at expense of considering the patient’s clinical seizure control and adverse effect monitoring. Blood levels may lead both physicians and patients to a false sense of therapeutic adequacy; for example, a patient still having breakthrough seizures on phenytoin with a blood level of 16 ug/mL might lead both the patient and physician to errantly believe that this patient was adequately treated on the drug, since the maintenance of a level in the “therapeutic” range could subjugate clinical judgment with a sense of complacency (or worse, acceptance of continued seizures as a necessary existence). On the other hand, levels may lead to errant manipulation of AEDs in patients who would otherwise require no adjustments; if a seizure-free patient had a phenytoin level measured of 6.0 ug/mL, and the clinician subsequently felt compelled to “treat the level” by increasing the daily phenytion dosage to raise the blood level into the usual “therapeutic range” of 10-20 ug/mL, this circumstance may lead to development of dose-related adverse effects in a patient who was already in an ideal clinical scenario (seizure free and without adverse effects).

A detailed discussion of AED safety monitoring is beyond the scope of this article, and was reviewed in the previous article in this series. Baseline safety monitoring at expense of considering the patient’s clinical seizure control and seizure control and adverse effect monitoring [12]. However, the extent and frequency of performing such monitoring remains unclear.

## CONCLUSIONS

There is no universally agreed upon standard or strategy for blood level monitoring during AED therapy. Blood levels are most useful as an adjunct to clinical wisdom to help meet the central goals for each patient: seizure freedom without side effects. When possible, employ AED monotherapy at the lowest effective dose, or perhaps a moderate target dose, and the time-honored maxim of “treating the patient, not the level” remains an excellent overall guiding principle. Optimization of AED therapy can be guided by blood level monitoring, and employing AED levels is especially beneficial for monitoring adherence, titrating AEDs in complex polypharmacy regimens, or adjusting for altered AED metabolism in disease states, puberty and aging, or pregnancy. However, AED blood levels should never replace clinical judgment, and over-reliance on AED levels may lead both physicians and patients to a false sense of therapeutic adequacy; no “therapeutic” level should suffice as evidence of adequate care when patients continue to experience breakthrough seizures or deleterious side effects.

## Figures and Tables

**Table 1. Common Pitfalls in AED Blood Level Monitoring and Dosing:** The table portrays pitfalls in AED blood level monitoring when the AED dosage is adjusted based on the level blindly, without regard to clinical efficacy or toxicity. Breakthrough seizures, inappropriate alterations in effective therapy, therapeutic complacency, and toxic side effects may result if the clinical considerations of efficacy and adverse effects are not given priority in adjustments to AED dosage.

AED Levels	Blind, Reactive AED Adjustment to Level	Patient’s Clinical Efficacy	Patient’s Clinical Side Effects	Resultant Pitfall
Supratherapeutic	Lower dose; move to new/next AED trial	Seizure-free	None	Breakthrough seizures; needless loss of ideal efficacy
Therapeutic	No	Intractable	None	Underdosing, therapeutic complacency
Subtherapeutic	Raise dose	Seizure-free	None/toxic	Toxic side effects possible

**Table 2.** Uses of AED Blood Level Monitoring

Setting	Rationale	Applicable AEDs	Usual AED Modification
**Assessing Complex Pharmacokinetics**	Narrow therapeutic Index; complex metabolism	Phenytoin above 300 mg/day; Carbamazepine	Variable: adjust phenytoin by small doses; reduce carbamazepine
**Initial Therapy**	Document successful therapy	All	Serves as “baseline” for future comparison if seizure control deteriorates
**During AED Conversions**	Ensure sufficiently protective dosing of new AED, Evaluate drug interactions	All	Raise or lower doses
**Therapeutic Failure**	Exclude non-adherence/non-compliance “rule-In” Intractability	All transition to new therapy; reinforce compliance	Raise dose or transition to new drug
**Pregnancy**	Altered pharmacokinetics	All	Raise or lower dose
**Liver/Renal Disease**	Altered AED metabolism/clearance	All	Usually lower dose
**Polypharmacy**	Altered pharmacokinetics	Enzyme-inducing AEDs; Enzyme-inhibiting AEDs; Inducible AEDs	Lower doses of inducing AED; Lower doses of other AED; Raise inducible AED dose
**Children/Adolescents**	Increased AED Metabolism/Clearance	Most	Raise doses
**Elderly**	Decreased AED Metabolism/Clearance	Many	Lower doses

**Table 3.** Common Doses and Levels of Older and Newer AEDs

	Adult Dosage/Day	Levels (uG/mL)
**Older AEDs**		
Carbamazepine	400-1600+ mg (600 mg)	4-12+
Ethosuximide	500-1500+ mg	40-100+
Phenobarbital	90-180+ mg	15-40
Phenytoin	200-400+ mg	8-20+
Primidone	500-1500+ mg	5-12 (measure phenobarbital)
Valproate	750-2500+ mg	50-100+
**Newer AEDs**		
Felbamate	1800-4800+ mg	30-100+
Gabapentin	900-3600+ mg	4-20++
Lacosamide	200-600+ mg	?
Lamotrigine	300-600+ mg	1-20+
Levetiracetam	1000-3000++ mg	5-40++
Oxcarbazepine	600-3600+ mg	10-40+ (measure MHD)
Rufinamide	400-3200 mg	?
Tiagabine	16-64 mg	100-300 ng/mL
Topiramate	100-600+ mg	10-20+
Zonisamide	100-600+ mg	10-40+

uG/mL= micrograms/milliliter.

+ = higher doses/levels often additionally effective, as tolerated.

++ = considerably higher doses/levels sometimes additionally effective in intractable patients, as tolerated.

MHD = 10, 11 Monohydroxyderivative active metabolite of oxcarbazepine.

nG/mL = nanograms/milliliter.

? = no evidence yet available to indicate typically therapeutic level since drug is newly approved.

**Table 4.** AED Adjustments in Context of Levels and Clinical Status

AED Levels	Efficacy	Side Effects	AED Adjustment?
Super therapeutic Or Sub therapeutic	Seizure-free	None	No
Super therapeutic Or Sub therapeutic	AED responder	Tolerable	Raise dose
Super therapeutic Or Sub therapeutic	Seizure-free	Intolerable	Lower dose; move to new/next AED trial
Super therapeutic Or Sub therapeutic	Intractable	Tolerable AEs	Raise dose if no response plateau
Super therapeutic Or Sub therapeutic	Intractable	Intolerable AEs	Lower dose; move to new/next AED trial; ?epilepsy surgery/VNS
Therapeutic	Seizure-free	No AEs	No

## References

[1] Booker H.E, Woodbury D.M, Penry J.K, Schmidt R.P (1972). Phenobarbital, mephobarbital, and metharbital: relation of plasma levels to clinical control. Antiepileptic Drugs.

[2] Chadwick D (1987). Overuse of monitoring of blood concentrations of antiepileptic drugs. BMJ.

[3] Gilliam FG, Fessler AJ, Baker G, Vahle V, Carter J, Attarian H (2004). Systematic screening allows reduction of adverse antiepileptic drug effects: a randomized trial. Neurology.

[4] Januzzi G, Cian P, Fattore C, Gatti G, Bartoli A, Monaco F, Perucca E (2000). A multicenter randomized controlled trial on the clinical impact of therapeutic drug monitoring in patients with newly diagnosed epilepsy. The Italian TDM Study Group in Epilepsy. Epilepsia.

[5] Kwan P, Brodie MJ (2001). Effectiveness of first antiepileptic drug. Epilepsia.

[6] Larkin JG, Herrick AL, McGuire GM, Percy-Robb IW, Brodie MJ (1991). Antiepileptic drug monitoring at the epilepsy clinic: a prospective evaluation. Epilepsia.

[7] Lesser RP, Pippenger CE, Luders H, Dinner DS (1984). High dose monotherapy in treatment of intractable seizures. Neurology.

[8] Schoenenberger RA, Tanasijevic MJ, Jha A, Bates DW (1995). Appropriateness of antiepileptic drug monitoring. JAMA.

[9] Sherwin AI, Robb JP, Woodbury DM, Penry JK, Schmidt RP (1972). Ethosuximide: relation of plasma levels to clinical control. Antiepileptic Drugs.

[10] Sherwin AI, Robb JP, Lechter M (1973). Improved control of epilepsy by monitoring ethosuximide. Arch. Neurol. (Chic).

[11] Theodore WH (1992). Rational use of antiepileptic drug levels. Pharmacol. Ther.

[12] Willmore LJ, Levy RH, Mattson RH, Meldrum BS, Perucca E (2002). Safety monitoring of antiepileptic drugs. Antiepileptic Drugs.

[13] Woo E, Chan YM, Yu YL, Chan YW, Huang CY (1988). If a well-stabilized epileptic patient has a subtherapeutic antiepileptic drug level, should the dose be increased? A randomized prospective study. Epilepsia.

[14] Yahr MD, Sciarra D, Carter S, Merritt HH (1952). Evaluation of standard anticonvulsant therapy in three hundred nineteen patients. J. Am. Med. Assoc.

